# Human herpes virus 6 or Epstein–Barr virus were not detected in Guthrie cards from children who later developed leukaemia

**DOI:** 10.1038/sj.bjc.6602099

**Published:** 2004-08-24

**Authors:** G Bogdanovic, Å G Jernberg, P Priftakis, L Grillner, B Gustafsson

**Affiliations:** 1 Department of Clinical Microbiology, Karolinska University Hospital Solna, Karolinska Institutet, S-171 76 Stockholm, Sweden; 2 Department of Paediatrics, Karolinska University Hospital Huddinge, Karolinska Institutet, S-141 86 Stockholm, Sweden; 3 Department of Oncology-Pathology, Cancer Centre, Karolinska University Hospital Solna, Karolinska Institutet, S-171 76 Stockholm, Sweden

**Keywords:** childhood ALL, Herpes virus, Guthrie cards, PCR, prenatal origin

## Abstract

To investigate if children who later developed acute lymphoblastic leukaemia (ALL) were prenatally infected with HHV-6 and/or EBV, Guthrie cards taken at birth were analysed by PCR. Guthrie cards from 54 patients with ALL and 47 healthy controls matched for age and birth place were tested negative for both HHV-6 and EBV DNA. All samples contained amplifiable DNA when tested by HLA-DQ PCR. Our negative findings suggest that childhood ALL is unlikely to be associated with an *in utero* infection with EBV or HHV-6.

Acute lymphoblastic leukaemia (ALL) is the most frequent childhood malignancy in Western countries. The different subtypes of leukaemia differ in their patterns of genetic change, natural history, prognosis and possibly aetiology (Doll, 1989; Ross, 1999). One model for the aetiology of ALL is prenatal infection and such a maternal infection could affect foetal haematopoiesis early in the liver and later in the bone marrow (Ohls and Christensen, 2000). The relevant agent would have to cross the placenta, induce genomic instability in the lymphocytes and infect the foetus without causing severe foetal abnormalities (Smith, 1997). We have analysed human polyomaviruses (JCV and BKV) and human parvovirus B19 in Guthrie cards from children who later developed ALL, but could detect neither polyomavirus DNA nor parvovirus DNA from ALL cases or controls (Priftakis *et al*, 2003; Isa *et al*, 2004).

Epstein–Barr virus (EBV) and human herpes virus 6 (HHV-6) are two potentially oncogenic viruses, widely distributed in the human population, which can be spread by vertical or horizontal (*in utero*) transmission. Since they persist and establish latency in their natural host after primary infection, they may be reactivated during immunosuppression (Matsuda *et al*, 1999). The viral latency is primarily established in lymphocytes, which may be important in relation to childhood ALL (Gustafsson *et al*, 2000; Salonen *et al*, 2002).

Our study was conducted to determine if a group of children with ALL were prenatally infected with HHV-6 or EBV. Guthrie cards taken at birth were collected and analysed for the presence of these viruses.

## PATIENTS AND METHODS

### Patients

We collected Guthrie cards from 54 children who had developed ALL. In all, 50 of these children had been diagnosed as pre-B ALL (either CD10+, CD20+, FAB L1 or L2) and four children were diagnosed as T-ALLs (CD3+ or CD8+). A total of 24 children were diagnosed before the age of 4, 16 children were between 5 and 9 years, 12 children were between 10 and 14 years of age and two children were older than 14 years. The median age at diagnosis was 5 years (range 9 months–17 years, mean 5.9 years). These children had been admitted for treatment at four different Paediatric Oncology Swedish Centres from 1980 through 2001. As a control group, we obtained 47 healthy controls that were matched for age and birthplace, and data from the controls were collected from the Swedish Medical Birth Register, which is a standardised set of medical records introduced in Sweden in 1973 (Cnattingius *et al*, 1990). The capillary blood from both groups is collected at 3–5 days of age. After the screening analysis, the Guthrie cards are stored at 4°C at the PKU laboratory, at Karolinska University Hospital, Huddinge.

Two bone marrow-transplanted patients were positive for HHV-6 and EBV-DNA, respectively. These two patients were included as positive controls. Guthrie cards ‘spiked’ with blood from these patients were tested for the presence of HHV-6 or EBV DNA. Blood samples were taken from these two patients and added to a new Guthrie card.

### Methods

#### DNA extraction by minimal essential medium (MEM)

At the time of analysis, the filter paper samples were stored at room temperature before extraction. The extraction method utilised was MEM extraction, previously described, with minor modifications (Barbi *et al*, 1996). Three uniform discs 3 mm in diameter, were removed from the filter paper, and prepared using special precautions in order to avoid contamination, as previously described.

#### HHV-6 DNA detection

Presence of HHV-6 DNA was analysed by a nested PCR, with the primers corresponding to the conserved region of HHV-6 of both variants A and B amplifying a 173 bp segment (Wang *et al*, 1996). Purified DNA extracted from HHV-6 strain GS (type A) and strain Z29 (type B) were used as positive controls in all PCRs. The sensitivity estimated using known concentrations of positive controls DNA was 20–30 genomes per PCR reaction for both strains (Wang *et al*, 1996).

#### EBV DNA detection

Presence of EBV DNA was analysed by a nested PCR with the primers corresponding to the fragment of EBV EBNA gene 1 amplifying a 208 bp segment (Cinque *et al*, 1993; Wang *et al*, 1996).

#### HLA PCR

As a control for the presence of DNA extracted from Guthrie cards and for DNA amplifiability, a PCR with a set of HLA DQ primers was performed (Saiki *et al*, 1986). The HLA PCR-negative samples were not used for further investigation.

### Ethical considerations

Written informed consent was obtained from patients, controls and parents. The local ethics committee of the Karolinska Institutet at Karolinska University Hospital approved the study protocol.

## RESULTS AND DISCUSSION

The Guthrie cards of all 54 patients with ALL, as well as all 47 healthy controls, were tested negative by PCR for both HHV-6 DNA and EBV DNA. All samples contained amplifiable DNA when tested by HLA-DQ PCR. HHV-6 or EBV DNA could be detected in the blood as well as in the ‘spiked’ Guthrie cards from the two BMT patients with HHV-6 and EBV infection, respectively.

The use of stored Guthrie cards as a source of DNA has been shown to have several uses. DNA is relatively stable and blood collected from newborns contains 2–3 times higher levels of nucleic acid when compared to adults. In several studies, it has been shown that viral DNA could be successfully amplified from Guthrie cards in case of congenital CMV, HSV and HIV infection (Cassol *et al*, 1991; Barbi *et al*, 1996; Fischler *et al*, 1999).

The difficulty to divide childhood leukaemia into homogenous subgroups may explain the complexity when investigating different aetiological factors. In our material, the majority of children had pre-B-ALL. It has been suggested that infection *in utero* with a virus that has the oncogenic potential could be involved in the initiation of ALL, by inducing genomic instability and allowing specific effects on B lymphocytes. Twin studies suggest that an additional molecular event or exposure is then required postnatally for the preleukaemic clone to expand. The second event, leading to leukaemia, can occur at a time of maximum stress on lymphocyte precursor proliferation and may be promoted by exposure to a common infectious agent (Gale *et al*, 1997; Greaves, 1999; Wiemels *et al*, 1999).

Space–time clustering of ALL has raised the possibility of an infectious agent in ALL. Kinlen and others proposed that the mixing of previously separate groups of people, as in the creation of new towns in rural construction projects or military camps, may raise the incidence of leukaemia by facilitating transmission of infective agents (Kinlen *et al*, 1990; Kinlen and Balkwill, 2001; Kinlen *et al*, 2002). Although most reports have emphasized space–time clustering at diagnosis (Petridou *et al*, 1996; Alexander *et al*, 1997; Gilman *et al*, 1999), recent studies have detected space–time clustering around the time of birth (Alexander, 1992; Gustafsson and Carstensen, 1999, 2000; Birch *et al*, 2000). This could implicate that events or exposures might originate *in utero*. But there are also opportunities for leukaemogenic infection to occur after birth and before the children in space–time clustering studies have moved away from birth addresses.

Another model for virus transmission during pregnancy is suggested by observations that leukaemia in cats can result from maternal transmission (Knox *et al*, 1980).

Intrauterine infection is a result of either primary maternal infection or reactivation of the latent infection. Primary infection with HHV-6 during pregnancy is a rare event, since more than 90% are seropositive at the age of 3, infection being most frequent during the first year of life (Dahl *et al*, 1990, 1999; Hall *et al*, 1994). HHV-6 reactivation may be more common when foetal transmission may occur in approx. 1% of pregnancies (Dahl *et al*, 1999). HHV-6 was first isolated from cultures of the peripheral blood lymphocytes of patients with AIDS and lymphoproliferative diseases; it is considered to play an important role in the development of complications after stem cell transplantation (Salahuddin *et al*, 1986; Matsuda *et al*, 1999). EBV was first discovered through its close relationship with the endemic form of Burkitt lymphoma, the most common childhood cancer in equatorial Africa (Burkitt, 1958; Epstein *et al*, 1964). EBV reactivation is also frequent in organ and stem cell transplant patients in whom it can produce lethal lymphoproliferative disease (Gustafsson *et al*, 2000). The experience of EBV in the transplant setting clearly illustrates the ability of the EBV virus to reactivate during immunosuppression and its oncological potential. Besides the transplant setting, EBV is also frequently reactivated during pregnancy (Costa *et al*, 1985). The seroprevalence of EBV in adults has been reported to be higher than 90% (Rickinson, 1996). In a recent published study (Lehtinen *et al*, 2003), it was shown that maternal reactivation of EBV infection during the first trimester was associated with a significant increased risk of developing ALL in the offspring. Maternal infection was determined by detection of EBV-specific IgM antibodies in the serum. As it is known, the specificity of IgM is not so good regarding EBV reactivation as compared to determining primary EBV infection, so PCR analysis was performed in addition (Lehtinen *et al*, 2003). Of 77 IgM positive women, there were only two that were EBV DNA positive, compared to three cases of EBV DNA-positive women in the control group, indicating that viremia was not often established during reactivation (Lehtinen *et al*, 2003).

The fact that neither HHV-6 DNA nor EBV were detected in dried blood spots obtained from newborns at birth indicates that congenital infections with these viruses are uncommon. The possibility of false-negative results due to infection early in pregnancy and being latent at birth must be considered. However, for congenital CMV infection, it has been shown that Guthrie cards were positive for CMV DNA even when the maternal infection took place during the first or second trimester, indicating that viremia persisted to birth (Peckham, 1991). Similar findings were reported in cases of intrauterine foetal death in association with parvovirus B19 infection (Tolfvenstam *et al*, 2001).

HHV-6 DNA and EBV-DNA were neither detected by PCR in blood from Guthrie cards in children who had developed ALL nor from healthy controls. Hence, it is unlikely that childhood ALL is associated with an *in utero* infection with these viruses although a latent infection cannot be ruled out by this method, nor infection after birth. In view of the epidemiological evidence for a relation between childhood ALL and infection, the search for a virus aetiology must continue.
